# Dynamics of Th17 Cells and Their Role in *Schistosoma japonicum* Infection in C57BL/6 Mice

**DOI:** 10.1371/journal.pntd.0001399

**Published:** 2011-11-15

**Authors:** Xiaoyun Wen, Lei He, Ying Chi, Sha Zhou, Jason Hoellwarth, Cui Zhang, Jifeng Zhu, Calvin Wu, Shawn Dhesi, Xuefeng Wang, Feng Liu, Chuan Su

**Affiliations:** 1 Department of Pathogen Biology and Immunology, Department of Pharmacology, Jiangsu Key Laboratory of Pathogen Biology, Nanjing Medical University, Nanjing, People's Republic of China; 2 Department of Educational Affairs, Keck School of Medicine, University of Southern California, Los Angeles, California, United States of America; University of Edinburgh, United Kingdom

## Abstract

**Background:**

The current knowledge of immunological responses to schistosomiasis, a major tropical helminthic disease, is insufficient, and a better understanding of these responses would support vaccine development or therapies to control granuloma-associated immunopathology. CD4^+^ T cells play critical roles in both host immune responses against parasitic infection and immunopathology in schistosomiasis. The induction of T helper (Th)1, Th2 and T regulatory (Treg) cells and their roles in schistosome infections are well-illustrated. However, little *in vivo* data are available on the dynamics of Th17 cells, another important CD4^+^ T cell subset, after *Schistosoma japonicum* infection or whether these cells and their defining IL-17 cytokine mediate host protective responses early in infection.

**Methodology:**

Levels of Th17 and the other three CD4^+^ T cell subpopulations and the cytokines related to induction or repression of Th17 cell generation in different stages of *S. japonicum* infection were observed. Contrary to reported *in vitro* studies, our results showed that the Th17 cells were induced along with the Th1, Th2, Treg cells and the IFN-γ and IL-4 cytokines in *S. japonicum* infected mice. The results also suggested that *S. japonicum* egg antigens but not adult worm antigens preferentially induced Th17 cell generation. Furthermore, decreasing IL-17 with a neutralizing anti-IL-17 monoclonal antibody (mAb) increased schistosome-specific antibody levels and partial protection against *S. japonicum* infection in mice.

**Conclusions:**

Our study is the first to report the dynamics of Th17 cells during *S. japonicum* infection and indicate that Th17 cell differentiation results from the integrated impact of inducing and suppressive factors promoted by the parasite. Importantly, our findings suggest that lower IL-17 levels may result in favorable host protective responses. This study significantly contributes to the understanding of immunity to schistosomiasis and may aid in developing interventions to protect hosts from infection or restrain immunopathology.

## Author Summary

Th17 immune cells secrete the IL-17 cytokine and contribute to host defenses against certain infections. Recent studies linked IL-17 with the severity of liver inflammation and suggested that Th17 cells contribute to the pathology in schistosomiasis, a serious disease caused by parasitic worms such as *Schistosoma japonicum* widespread in vertebrates including humans. However, the role of Th17 cells in protection against *S. japonicum* infection is still unclear. For the first time, we describe here the changes in Th17 cell levels during *S. japonicum* infection and suggest that the schistosome egg antigens are primarily responsible for stimulating the generation of host Th17 cells after *S. japonicum* infection. We further show that the level of Th17 cells in the host is determined by a combination of factors, namely exposure to complex parasitic antigens that either induce or suppress their generation. We also suggest that lowering IL-17 levels may favor the host's protective responses against *S. japonicum* infection. Our findings help to better understand the relationship between the host and parasite in terms of immune protection and pathology in schistosomiasis and may contribute to the future development of vaccination and therapeutic strategies.

## Introduction

CD4^+^ T cells play an important role in the initiation of immune responses against an infection by providing help to other cells and by taking on a variety of effector functions during immune reactions. Upon antigenic stimulation, naive CD4^+^ T cells activate, expand and differentiate into different effector subsets termed T helper (Th) 1 and Th2 cells. The appropriate induction and balance between Th1 and Th2 cellular responses to an infectious agent can influence both pathogen growth and immunopathology [1]. Th17 cells recently emerged as a third independent effector cell subset differentiated from CD4^+^ T cells upon antigenic stimulation [2]–[5]. Although the functions of these cell subtypes are not completely understood, emerging data suggest that by producing their defining cytokine IL-17, Th17 cells play an important role in host defenses against extracellular pathogens, such as *Klebsiella pneumoniae* [6], *Pseudomonas aeruginosa* [7], *Porphyromonas gingivalis* [8] and *Bacteroides fragilis* [9], which are not efficiently cleared by Th1-type and Th2-type immunity. Meanwhile, several studies have shown that Th17 cells and IL-17 also play important roles in immunopathology in some infectious diseases, such as pulmonary tuberculosis [10], toxoplasmosis [11] and schistosomiasis [12]–[17].

CD4^+^ T cells can also be induced to differentiate into CD4^+^CD25^+^ T regulatory (Treg) cells with immunosuppressive activities that down-regulate immune responses, thereby inhibiting immunopathology while promoting parasite survival via direct repression of the induction and responses of the other CD4^+^ subsets, Th1, Th2 and Th17 cells [18]–[22].

Since the functional analysis of IL-17 produced by Th17 cells has suggested an important and unique role for this cytokine in both host protection against specific pathogens and immunopathologic damage to the host, much of the research focus has been placed on the factors that either positively or negatively regulate differentiation of Th17 cells. To date, several studies have shown that Th17 cells require specific cytokines for their differentiation, different from those for Th1 and Th2 cells. A combination of TGF-β plus IL-6 was recently described to be essential for initial differentiation [23]–[27], IL-21 for the amplification [28], [29] and IL-23 for the subsequent stabilization [25], [30], [31] of the Th17 cell subset. On the other hand, both high levels of Th1 and Th2 cells and their respective cytokines, IFN-γ and IL-4, antagonize the development of Th17 cells [2], [4], [5]. Additionally, in the absence of IL-6, TGF-β alone is clearly favored as the cytokine for differentiation of Treg cells while suppressing the differentiation of Th17 cells [4], [32]. These findings suggest an intimate link between the Treg and Th17 cell programs of differentiation. However, thus far the notion that CD4^+^ T cell subsets represent distinct terminally differentiated lineages has been favored on the basis of a series of *in vitro* experiments, and the suppression of Th17 differentiation by Th1, Th2 and Treg cells and/or their cytokines has been demonstrated in numerous *in vitro* studies or under certain simplified or defined conditions [25], [27], [32]–[34]. However, there is very little *in vivo* data available to support such a cross-regulation between Th17 cell differentiation and Th1, Th2 and Treg cells during multicellular pathogenic infection.

Schistosomiasis, a major neglected tropical helminthic disease infecting 200 million people with an estimated 600 million at risk worldwide, is an excellent model for studying the induction and regulation of differentiation of the various CD4^+^ T cell subsets in response to infection. Infection of *Schistosoma japonicum*, a multicellular parasite which has an extremely diverse repertoire of antigens, induces the production of bulk cytokines to induce Th1, Th2 and Treg cells that play important roles in the immune response to infection. In particular, a recently growing number of studies have indicated that IL-17, a CD4^+^ T cell-derived cytokine, is most directly associated with the severity of hepatic granulomatous inflammation [12]–[16], [35]–[37], suggesting that IL-17-producing T cells are a major force behind severe pathology in schistosomiasis. During schistosome infection, the immune response progresses through at least three phases. (1) During the first three weeks of the infection, when the host is exposed to migrating immature and mature parasites, the dominant response is Th1-like. The response is induced by non-egg antigens, such as the schistosomula and soluble worm antigen (SWA) [38], [39]. (2) As the parasites begin to produce eggs (beginning 4–5 weeks post-infection), the response alters, with the emergence of a stronger Th2 response which is primarily induced by egg antigens [38], [40]. The granulomas that form around the eggs in the liver, which are reported to be positively regulated by Th17 cells and the secreted IL17 cytokine [12]–[16], [36], develop to their maximum size around 8–9 weeks post-infection. (3) During the chronic phase of infection (beginning 11–13 weeks post-infection), the Th2 response is predominant and modulated. The granulomas are also smaller than at earlier times. At this stage, CD4^+^CD25^+^Foxp3^+^ Treg cells are believed to be induced mainly by egg antigens and play an important repressor role in down-regulation of pathologic immune responses [20]. In addition, a recent study reported elevated Th17 levels in response in vaccination against *S. mansoni* infection in C57BL/6 mice [41]. However, there is very little data available showing the dynamics of Th17 cells after *S. japonicum* infection as well as whether Th17 cells/IL-17 mediate the host protective responses at the early stage of *S. japonicum* infection. In the present study, we observed the changes in Th17 cell levels at different stages of *S. japonicum* infection and investigated the role of IL-17 in the host protective responses.

## Materials and Methods

### Ethics statement

Animal experiments were performed in strict accordance with the Regulations for the Administration of Affairs Concerning Experimental Animals (1988.11.1), and all efforts were made to minimize suffering. All animal procedures were approved by the Institutional Animal Care and Use Committee (IACUC) of Nanjing Medical University for the use of laboratory animals (Permit Number: NJMU 07-0137).

### Mice, parasites and infection

Female 8-week old C57BL/6 mice were purchased from SLAC Laboratory (Shanghai, China) and bred in university facilities. All animal experiments were performed in accordance with the Chinese laws for animal protection and in adherence to experimental guidelines and procedures approved by the Institutional Animal Care and Use Committee (IACUC), the ethical review committee of Nanjing Medical University, for the use of laboratory animals. *Oncomelania hupensis* harboring *S. japonicum* cercariae (Chinese mainland strain) were purchased from the Jiangxi Institute of Parasitic Diseases (Nanchang, China).

For kinetic analysis of T cell populations and cytokines, each mouse was infected with 12 cercariae of *S. japonicum* through the abdominal skin. At 3, 5, 8 and 13 weeks post-infection, four mice were randomly chosen from the infected and normal control groups and sacrificed for further study. For challenge experiments, each mouse was infected with 40 cercariae of *S. japonicum* by abdominal skin exposure.

### Preparation of antigens and immunization schedule

*S. japonicum* SWA were prepared by harvesting the soluble fraction obtained from sonicated *S. japonicum* adult worms as previously described [42]. *S. japonicum* eggs were extracted from the livers of infected rabbits and enriched. The *S. japonicum* soluble egg antigens (SEA) were then prepared from the homogenized eggs as previously described [43]. SWA and SEA were diluted with PBS to a final concentration of 10 mg/ml for immunization.

Three independent experiments were carried out in the same manner. In each experiment, C57BL/6 mice were divided randomly into three groups (two test and one control) consisting of eight mice per group. Each mouse was injected subcutaneously in the back with 100 µl of a solution containing 50 µg of SEA, 50 µg of SWA or PBS emulsified in incomplete Freund's adjuvant (IFA, Sigma-Aldrich, ST. Louis, MO) [44]. Each mouse was immunized two times with a 14-day interval. Two weeks after the last immunization, serum samples were collected, and mice were sacrificed for further study.

### Cell culture

Single cell suspensions of splenocytes and lymphocytes were prepared by mincing the mouse spleens and mesenteric lymph nodes in PBS containing 1% FBS (Gibco, Grand Island, NY) and 1% EDTA. Red blood cells were lysed using ACK lysis buffer.

For preparation of single cell suspensions of hepatic lymphocytes, mouse livers were perfused via the portal vein with a PBS/heparin mixture (75 U/ml, Sigma Chemical Co., St. Louis, MO). The excised liver was cut into small pieces and incubated in 10 ml of digestion buffer (collagenase IV/dispase mix, Invitrogen Life Technologies, Carlsbad, CA) for 30 min at 37°C. The digested liver tissue was then homogenized using a MediMachine with 50 µm Medicons (Becton Dickinson, San Jose, CA) for 3 min at low speed [45]. The liver suspension was then centrifuged at low speed to sediment the hepatocytes. The remaining cells were separated on a 35% Percoll gradient by centrifuging at 600×*g*. The lymphocyte fraction was resuspended in 2 ml of red cell lysis buffer and then washed in 10 ml of complete RPMI 1640 with 0.1 M EDTA.

The cells were cultured in triplicate in complete RPMI 1640 medium (Gibco) containing 10% FBS, 2 mM pyruvate, 0.05 mM 2-mercaptoethanol, 2 mM L-glutamine, 100 U of penicillin/ml and 0.1 mg/ml streptomycin. Subsequently, 2×10^5^ cells per well in 200 µl of complete media were cultured in 96 well plates (Nunc, Roskilde, Denmark) for 72 h at 37°C in the presence of 25 ng/ml phorbol 12-myristate 13-acetate (PMA) and 1 µg/ml ionomycin (Sigma-Aldrich) [46]–[48]. Alternatively, in some experiments, the cells from *S. japonicum* infected mice were stimulated with or without 50 µg/ml of SEA for 48 h. Culture supernatants were collected for ELISA after incubation.

### Cytokine and antibody detection

Cytokines in the culture supernatant were analyzed using mouse cytokine multiplex assay kits for detecting IL-6 and IL-21 (R&D Systems, Inc. Minneapolis, MN) and for detecting TGF-β and IL-23 (Bender MedSystems, Novato, CA). IL-17A, IFN-γ and IL-4 levels in the supernatant were measured by ELISA using the eBioscience ELISA Ready-SET-Go kit (eBioscience, San Diego, CA) according to the manufacturer's protocol.

The SWA and SEA specific IgG, IgG1 and IgG2a antibodies in mouse serum samples were detected by standard ELISA using the SWA and SEA as the coated antigen [42], [43]. HRP-conjugated rat anti-mouse IgG (Calbiochem, Darmstadt, Germany), IgG1 and IgG2a monoclonal antibodies (mAbs) (BD Pharmingen) were used. In brief, ELISA plates (Titertek Immuno Assay-Plate, ICN Biomedicals Inc., Costa Mesa, CA) were coated with 0.1 mg/ml of SEA or SWA in 50 mM carbonate buffer (pH 9.6) and incubated overnight at 4°C. Plates were washed three times with PBS (pH 7.6) containing 0.05% Tween-20 (PBS-T) and blocked with 0.3% (w/v) bovine serum albumin (BSA) in PBS for 1 h at 37°C. The plates were further washed three times with PBS-T and then incubated with the sera diluted with 0.3% BSA (1∶100) at 37°C for 1 h. The plates were washed four times with PBS-T, followed by incubation with HRP-conjugated rat anti-mouse IgG, IgG1 and IgG2a (1∶1000) for 1 h at 37°C. The plates were then washed five times with PBS-T and developed with tetramethylbenzidine (TMB) substrate (BD Pharmigen) for 30 min. The optical density (OD) of the color developed in the plate was read at 450 nm using a BioRad (Hercules, CA) ELISA reader.

### Intracellular cytokine and Foxp3 staining

For detection of Th17, Th1 or Th2 cells, single cell suspensions of splenocytes, lymphocytes or liver cells from each mouse were prepared, and 1×10^6^ cells from each sample were stimulated with 25 ng/ml PMA and 1 µg/ml ionomycin (Sigma-Aldrich) in complete RPMI 1640 medium in the presence of 0.66 µl/ml Golgistop (BD Biosciences PharMingen) for 6 h at 37°C in 5% CO_2_. After 6 h, the cells were collected and surface stained with anti-CD3-APC (eBioscience) and anti-CD4-FITC (eBioscience). Subsequently, the cells were washed, fixed, permeabilized with Cytofix/Cytoperm buffer (BD PharMingen) and intracellularly stained with PE conjugated antibodies against IL-17A, IFN-γ or IL-4 (or isotype IgG2a control antibody) (eBioscience) for detection of Th17, Th1 or Th2 cells, respectively, according to the manufacturer's protocol and analyzed with a FACS Calibur flow cytometer. Cells were gated on the CD3^+^CD8^−^ population for analysis of Th17, Th1 or Th2 cells.

For detection of Treg cells, the Mouse Regulatory T Cell Staining Kit (eBioscience) was used. A single cell suspension of splenocytes from each mouse was prepared, and 1×10^6^ cells were surface stained with anti-CD3-PerCP mAbs (eBioscience), anti-CD4-FITC mAbs and anti-CD25-APC mAbs, followed by fixation and permeabilization with Cytofix/Cytoperm and intracellular staining with anti-Foxp3-PE or IgG2a-PE rat immunoglobulin control antibody, according to the manufacturer's protocol. Cells were gated on the CD3^+^CD4^+^ population for analysis of Treg cells.

### rmIL-17A and neutralizing anti-mouse IL-17A mAb administration and *S. japonicum* infection

The recombinant mouse IL-17A (rmIL-17A), the neutralizing rat anti-mouse IL-17A mAb (clone 50104) and its control IgG2a mAb were purchased from R&D Systems, Inc. Two independent experiments were carried out in the same manner. In each experiment, 16 mice were randomly assigned in four groups (four mice per group). Each mouse was challenged with 40 cercariae of *S. japonicum* as described above. For two groups of mice, 70 µg of mAb or its control IgG2a mAb per mouse were administered intraperitoneally (i.p.) four days before S. *japonicum* infection, and the administration of the same dose of mAb was repeated every four days during the infection until two days before the mice were sacrificed [12]. Simultaneously, for the other two groups of mice, 500 ng/mice of rmIL-17A or PBS were administered i.p. two days before S. *japonicum* infection and repeated every 48 h during the infection until two days before the mice were sacrificed [49]. Forty-two days after the challenge infection (two days after the last injection of rmIL-17A or anti-IL-17A), all four mice in each group were sacrificed. Serum samples were collected for ELISA detection of the levels of SEA or SWA specific antibodies, and the livers were isolated for histopathological examination. The splenocytes were prepared for incubation as previously mentioned for detection of cytokines in the culture supernatant or intracellular staining for detection of Th17, Th1, Th2 and Treg cells.

### Histopathological examination and estimation of worm and egg burdens in liver

Forty-two days after the challenge, all mice injected with neutralizing mAb or rmIL-17A and their controls were sacrificed, and perfusion was performed with saline containing heparin to recover the adult worms. Two grams of each liver were digested with 5% KOH at 37°C overnight, and the numbers of eggs were determined by microscopic examination. The remaining parts of the livers were dissected and immediately fixed in 10% buffered formalin. Liver sections were embedded in paraffin and stained with hematoxylin and eosin (H&E) for microscopic examination. The lesions were assessed on coded slides by an observer unaware of the experimental setting. The sizes of the granulomas were measured by computer-assisted morphometric analysis as previously described [44], and 50 visual fields in the liver section of each mouse (ten sections for each mouse and five random microscope fields for each section) were measured under a microscope (magnification: 100×) (Olympus, Tokyo, Japan). Granuloma sizes are expressed as means of areas measured in µm^2^ ± SD. The percentages of neutrophils, eosinophils, lymphocytes and macrophages in the same granulomas were determined by microscopic examination (1000× magnification) of 200 randomly selected cells (not including hepatocytes) in each granuloma. Ten sections for each mouse and five microscope fields for each section were counted. Percentages of cells were calculated from microscopic analysis of the same granulomas analyzed for lesion size [14], [50], [51].

The worm/egg reduction rate (percentage of protection) was calculated according to the following formula: (1 - mean of worms/eggs in injected mice/mean of worms/eggs in control mice)×100% [52].

### Statistical analysis

Statistical analysis was performed using the SPSS version 10.1 (Statistical Package for Social Sciences, Chicago, IL) software. Statistical significance was determined by Student's *t*-test and *P*<0.05 was considered significant.

## Results

### Kinetic analysis of the proportions of Th17, Th1, Th2 and Treg cells among CD4^+^ T cells during *S. japonicum* infection in C57BL/6 mice

**Figure 1:**
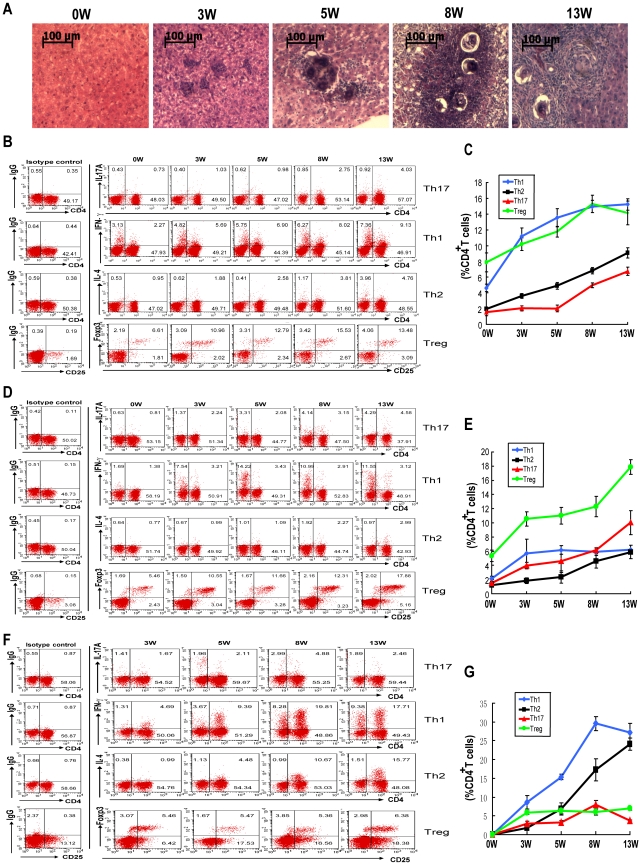
Kinetics of Th17, Th1, Th2 and Treg cell induction in *S. japonicum* infection. For each of three independent experiments, 20 female C57BL/6 mice were infected with 12 cercariae of *S. japonicum* per mouse. Four mice were randomly chosen and sacrificed at 0 (before infection), 3, 5, 8 or 13 weeks post-infection. Liver sections were stained with H&E for microscopic examination. Single cell suspensions of splenocytes, lymphocytes or liver cells were stimulated with PMA and ionomycin in the presence of Golgistop for 6 h. Cells were surface stained with anti-CD3-APC and anti-CD4-FITC and then intracellularly stained with PE-conjugated antibodies against IL-17A, IFN-γ, IL-4 or isotype IgG2a control antibody for FACS analysis of Th17, Th1 or Th2 cells. Tregs were detected in single cell suspensions of splenocytes, lymphocytes or liver cells using the Mouse Regulatory T Cell Staining Kit. **A.** Histopathology in the livers (magnification: 400×). Results are representative of three independent experiments. The kinetics of the percentages of Th17, Th1, Th2 and Treg cells in total CD4^+^ T cells from mouse spleens (**B**), mesenteric lymph nodes (**D**) and livers (**F**). Data are expressed as the mean ± SD of 12 mice from three independent experiments. Flow cytometric analysis of CD4^+^ T cell subsets in mouse splenocytes (**C**), mesenteric lymphocytes (**E**) and hepatocytes (**G**) from one representative experiment. Cells were gated on the CD3^+^ population for analysis of Th17, Th1, Th2 cells, or gated on the CD3^+^CD4^+^ population for analysis of Treg cells.

Consistent with previous studies, the granulomas began to form from five weeks after infection in the mouse liver after egg deposition and continued to develop (Fig. 1A). As shown in Fig. 1B and 1C, in parallel with the development of the granulomas, the proportion of Th17 cells in splenic CD4^+^ T cells increased very slowly during the first five weeks post-infection compared to that before infection (week 0) and increased rapidly thereafter. Meanwhile, the proportion of the Treg cells in the total splenic CD4^+^ T cell population showed a continuous increase after infection. Additionally, the proportions of both Th1 and Th2 cells in CD4^+^ T cells also increased. During the first three weeks post-infection, the proportion of Th1 cells rose much more quickly than that of the Th2 cells. However, after egg deposition, the number of Th2 cells kept increasing rapidly, while the number of Th1 cells reached a plateau by eight weeks post-infection (Fig. 1B and 1C). Compared to the CD4^+^T cells responses in the spleen, that of the mesenteric lymphocytes showed a more rapid increase of Th17 cells during the first three weeks post-infection and a weaker Th1 response throughout infection (Fig. 1D and 1E). Meanwhile, stronger Th2 but weaker Treg responses were observed in the liver (Fig. 1F and 1G). These results indicated that all of the CD4^+^ T cell subsets (Th17, Th1, Th2 and Treg cells) increased as over the course of infection.

### ELISA detection of cytokines in the supernatant of cultured splenocytes

**Figure 2:**
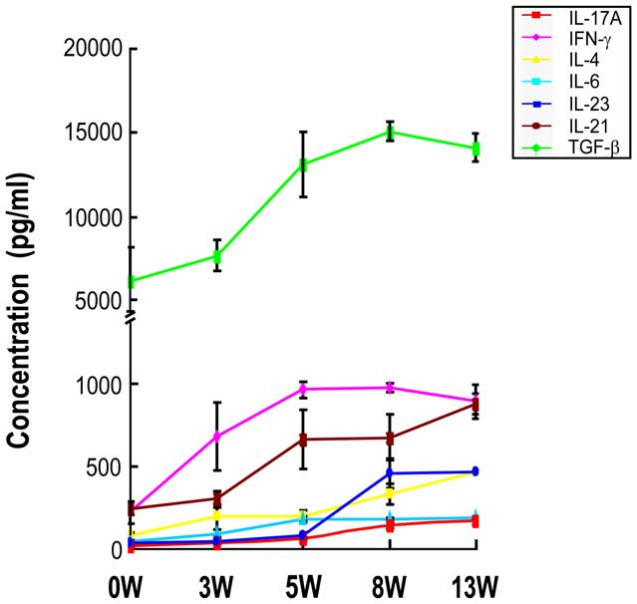
Kinetics of cytokines in the supernatant produced by splenocytes from *S. japonicum* infected mice. For each of three independent experiments, 20 female C57BL/6 mice were infected with 12 cercariae of *S. japonicum* per mouse. At 0 (before infection), 3, 5, 8 and 13 weeks post-infection, four mice at each time point were randomly chosen and sacrificed. Single cell suspensions of splenocytes were prepared and then cultured in the presence of PMA and ionomycin. The culture supernatants were collected after 72 h of incubation for detection of IL-17, IFN-γ, IL-4, TGF-β, IL-6, IL-23 and IL-21 by ELISA. Data are expressed as the mean ± SD of 12 mice from three independent experiments.

To further investigate the kinetics of cytokines which affect the differentiation, development and proliferation of Th17 cells during *S. japonicum* infection in C57BL/6 mice, the splenocytes of infected mice were cultured, and the cytokine levels in the supernatants were detected by ELISA. The results in Figure 2 show that, consistent with the generation of Th17 cells, the level of IL-17 increased very slowly in the first five weeks post-infection compared to that before infection (week 0). However, IL-17 increased rapidly after five weeks post-infection. Meanwhile, both the inducing cytokines (TGF-β, IL-6, IL-23 and IL-21) and the inhibitory cytokines (IFN-γ and IL-4) of Th17 cell generation all increased after infection. Taken together, these results suggest that the generation of Th17 cells during infection with *S. japonicum* may occur as a net effect of the inducing and inhibitory factors.

### FACS analysis of the proportions of Th17, Th1, Th2 and Treg cells in CD4^+^ T cells in mice immunized with SWA or SEA

**Figure 3:**
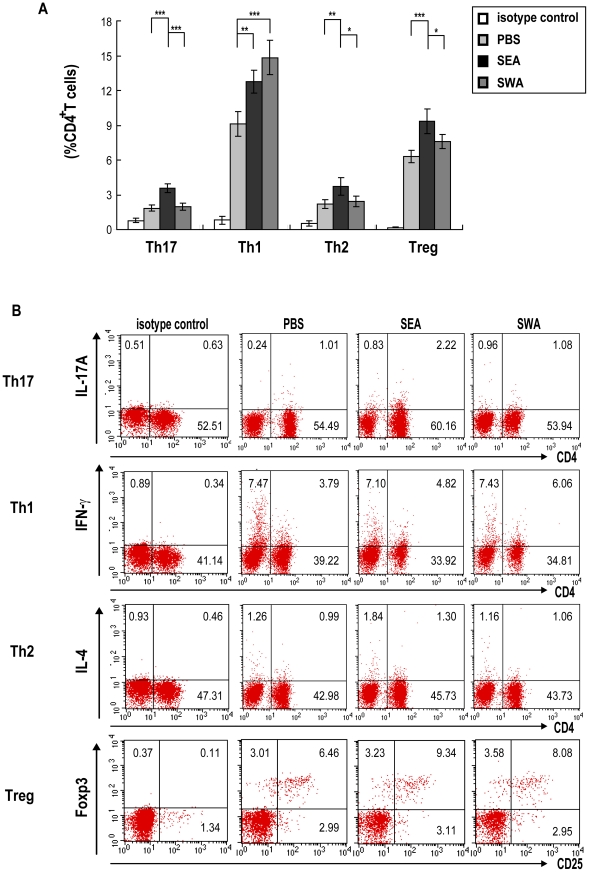
Proportions of Th17, Th1, Th2 and Treg cells in mice immunized with SEA or SWA. For each of three independent experiments, C57BL/6 mice (8 per group) were each injected subcutaneously in the back with 100 µl of an IFA emulsified solution containing 50 µg of SEA, 50 µg of SWA or PBS. The immunizations were repeated two times with a 14-day interval. Two weeks after the final vaccination, the mice were sacrificed, and single cell suspensions of splenocytes were prepared. The splenocytes were stimulated with PMA/ionomycin in the presence of Golgistop for 6 h, followed by surface staining with anti-CD3-APC and anti-CD8-FITC and then intracellular staining with PE-conjugated antibodies against IL-17A, IFN-γ, IL-4 or isotype IgG2a control antibody for FACS analysis of Th17, Th1 or Th2 cells. Splenocytes were stained with the Mouse Regulatory T Cell Staining Kit for detection of Treg cells. **A.** Proportions of Th17, Th1, Th2 and Treg cells in CD4^+^ T cells. Results are expressed as mean ± SD of 24 mice from 3 independent experiments. *P<0.05; **P<0.01; ***P<0.001, compared to control mice injected with PBS. **B.** Flow cytometric analysis from one representative experiment. Cells were gated on the CD3^+^ population for analysis of Th17, Th1 and Th2 cells or on CD3^+^CD4^+^ population for analysis of Treg cells.

Schistosome parasitic worms are multicellular pathogens which have three different life cycle stages (schistosomula, adult worm and egg) in definitive hosts including humans. Among the multitude of schistosome antigens that stimulate host immune responses, the adult worm and egg are two important sources of antigens that are involved induction of different types of Th cell responses or Tregs at different infection stages. Studies show that *Schistosoma mansoni* eggs induce egg antigen-specific Th17 responses and contribute to the severe immunopathology in murine schistosomiasis [12]–[17]. Consistent with these studies, our data also suggest that the *S. japonicum* egg antigens have the ability to significantly induce egg antigen-specific Th17 responses (Figure S1A, S1B and S1C). To further investigate the roles of these two major types of *S. japonicum* antigens on Th17 cell generation, SWA and SEA were used to immunize C57BL/6 mice or to induce CD4^+^T cells to differentiate *in vitro*. As shown in Figure 3A and 3B, a significantly higher percentage of Th17 cells was only observed in the SEA immunized group by FACS analysis, suggesting that repeated vaccinations of mice with SEA, instead of SWA, preferentially induced Th17 cells *in vivo*. Furthermore, the data also suggests that the eggs produced by adult worms in hosts, compared to the adult worms themselves, may more rapidly induce Th17 cells during *S. japonicum* infection. Meanwhile, additionally, SEA preferentially induced a significant increase of Th2 cells and Tregs, while SWA preferentially induced a significant increase of Th1 cells and caused only a slight increase of Treg and not of Th17 or Th2 cells (Figure 3A and 3B). The profiles of CD4^+^ T cells differentiation induced by SWA or SEA stimulation *in vitro* also confirmed the above findings (Figure S2A and S2B).

### ELISA detection of cytokines secreted in culture by splenocytes from SEA or SWA immunized mice

**Figure 4:**
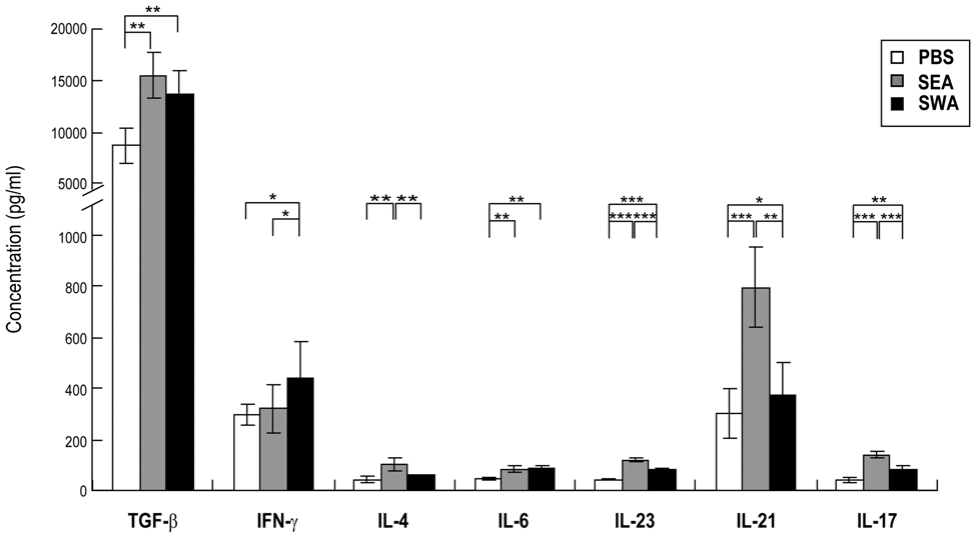
ELISA detection of cytokines in the supernatant produced by SEA or SWA immunized mice splenocytes. For each of three independent experiments, C57BL/6 mice (8 per group) were each injected subcutaneously in the back with 100 µl of an IFA emulsified solution containing 50 µg of SEA, 50 µg of SWA or PBS. The immunizations were repeated two times with a 14-day interval. Two weeks after the final vaccination, the mice were sacrificed, and single cell suspensions of splenocytes were prepared. The splenocytes (2×10^5^/well in 200 µl complete media) were cultured in 96 well plates with PMA/ionomycin. Culture supernatants were collected after 72 h, and cytokines were detected by ELISA. Results are expressed as mean ± SD of 24 mice from three independent experiments. *P<0.05; **P<0.01; ***P<0.001, compared to control groups as indicated.

To further investigate the cytokines that were reported to affect the differentiation, development and proliferation of Th17 cells, splenocytes from mice after vaccination with SWA or SEA were isolated and cultured as described in Materials and Methods, and the levels of cytokines in the supernatants were detected by ELISA. As shown in Figure 4, compared to the SWA and PBS control groups, a significantly higher level of IL-17 was observed in the SEA group, suggesting that repeated vaccination with SEA preferentially induced the production of IL-17. Compared to the PBS control group, the increase of IL-4 was mainly observed in the SEA group, while the increase of IFN-γ was only observed in the SWA group. Compared to the PBS control group, the levels of TGF-β, IL-6, IL-23 and IL-21, which are associated with the generation of Th17 cells, were significantly increased in both the SEA and SWA groups. However, when comparing the SWA group with the SEA group, the data showed that repeated vaccination with SEA preferentially induced higher levels of IL-23 and IL-21, and further supports that egg antigens are possibly more important in the increase of Th17 cells during *S. japonicum* infection.

### Protection against *S. japonicum* infection by administration of recombinant mouse (rm) IL-17A or anti-IL-17A neutralizing mAb in C57BL/6 mice

**Figure 5:**
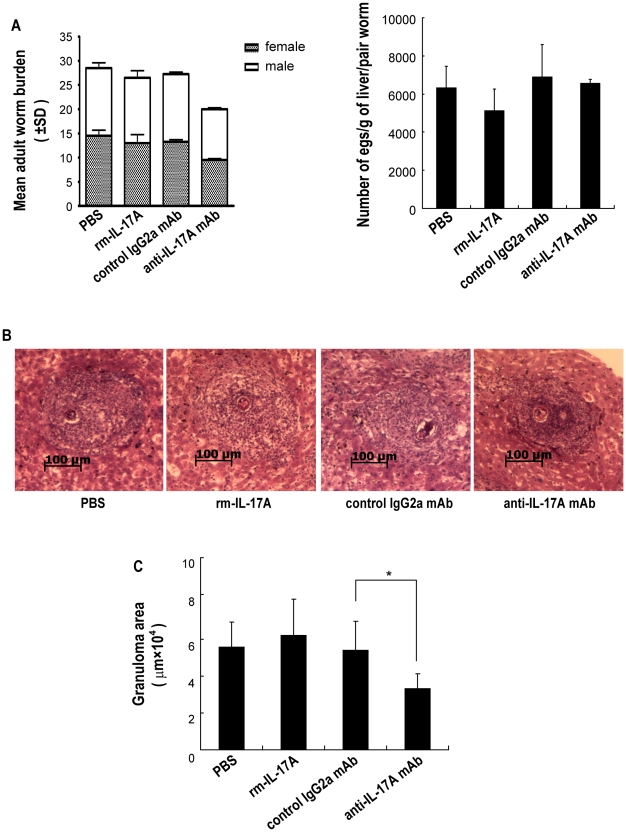
Protection against *S. japonicum* infection in C57BL/6 mice administered rmIL-17A or anti-IL-17A neutralizing mAb. rmIL-17A, anti-mouse IL-17A mAb or controls were administered to mice, followed by challenge with 40 *S. japonicum* cercariae per mouse. **A.** Six weeks after the challenge, the mice were sacrificed and perfused to collect adult worms. The numbers of eggs extracted from the liver were determined by microscopic examination. Protection was measured by assessing the worm and egg burden. Values are given as mean ± SD of eight mice from two independent experiments. *P<0.05; **P<0.01; ***P<0.001, compared to control mice injected with isotype antibody. **B.** Paraffin embedded formalin fixed liver sections stained with H&E. Images shown are representative of two independent experiments. **C.** Sizes of the granulomas were measured by computer-assisted morphometric analysis. Ten sections for each mouse and five microscope fields for each section were measured under a microscope. Only granulomas with a visible central egg, which reflected their true shape and dimension, were analyzed for accuracy. Values are given as mean ± SD of eight mice from two independent experiments. *P<0.05; **P<0.01; ***P<0.001, compared to control mice injected with isotype antibody.

To evaluate the role of IL-17 in the host protective responses against *S. japonicum* infection, C57BL/6 mice were injected with rmIL-17A or anti-IL-17A neutralizing mAb to increase or decrease the level of IL-17 *in vivo*, respectively, and then challenged with *S. japonicum*. The protection was measured by the reduction in the worm and egg burden [52] compared between groups injected with rmIL-17A or anti-IL-17A neutralizing mAb and their respective controls. Compared to the control IgG2a mAb group, injection of mice with neutralizing anti-IL-17A mAb led to a 26.61% reduction (*P*<0.01) in worm burden (Fig. 5A). On the other hand, compared to the PBS control group, no reduction of worm (*P*>0.05) or egg (*P*>0.05) burden was observed in the rmIL-17A group.

**Figure 6:**
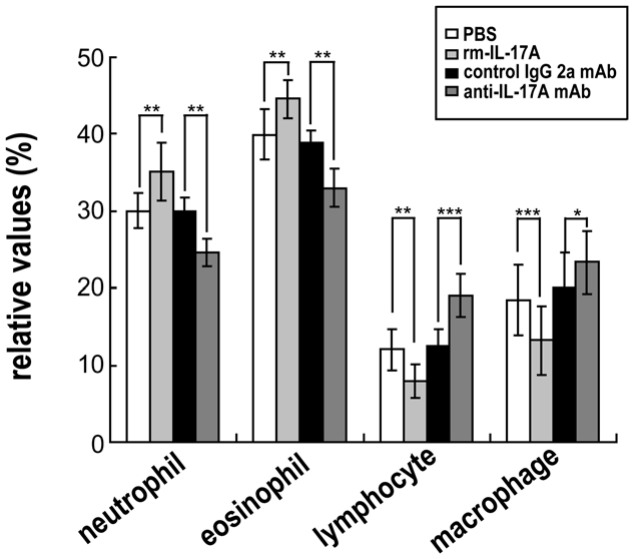
Percentages of granulomatous cells in *S. japonicum* infected mice administered rmIL-17A or anti-IL-17A neutralizing mAb. rmIL-17A, anti-mouse IL-17A mAb or controls were administered to mice, followed by challenge with 40 *S. japonicum* cercariae per mouse. Six weeks after the challenge, the mice were sacrificed, and the livers were processed for H&E staining. Percentages of neutrophils, eosinophils, lymphocytes and macrophages in the granulomas were calculated by microscopic analysis of the same granulomas analyzed for lesion size by randomly counting 200 cells (not including hepatocytes) in each granuloma. Ten sections for each mouse and five microscope fields for each section were counted. Values are given as mean ± SD of eight mice from two independent experiments. *P<0.05; **P<0.01; ***P<0.001, compared to control mice injected with PBS or isotype antibody.

Consistent with other reports [12]–[16], [36], our results in Figure 5B and 5C also show that elevating IL-17 *in vivo* by injecting mice with rmIL-17A led to slightly enhanced hepatic granulomatous inflammation (without statistical significance), while decreasing the level of IL-17 by use of an anti-IL-17A neutralizing mAb led to decreased hepatic immunopathology. The percentages of neutrophils and eosinophils in the granulomas, which are thought to be the important populations [14], were increased when *S. japonicum* infected mice were injected with rmIL-17A. However, injection with an anti-IL-17A neutralizing mAb led to decreases in the percentages of neutrophils and eosinophils in the granulomas (Fig. 6). In addition, the results in Figure 6 also show that rmIL-17A decreased while anti-IL-17A neutralizing mAb increased the percentages of lymphocytes and macrophages in *S. japonicum* infected mice.

### Effect of rmIL-17A or anti-IL-17A neutralizing mAb on CD4^+^T cells, cytokines and antibody responses in *S. japonicum* infected mice

**Figure 7:**
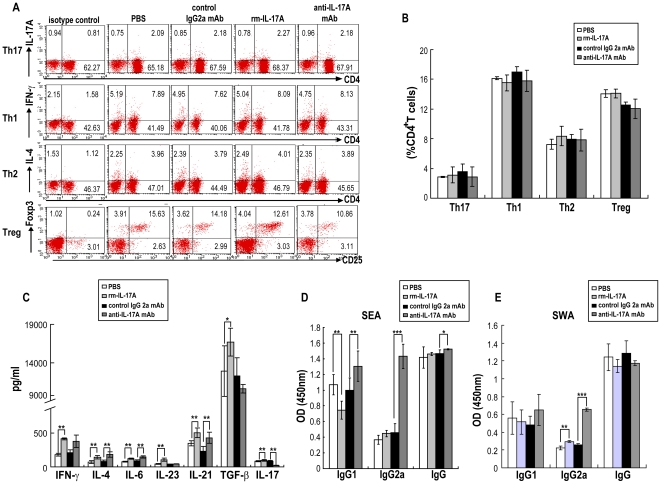
Immune responses in *S. japonicum* infected mice after administration of rmIL-17A or anti-IL-17A neutralizing mAb. rmIL-17A, anti-mouse IL-17A mAb or controls were administered to mice, followed by challenge with 40 *S. japonicum* per mouse. Six weeks after the challenge, the mice were sacrificed, and the splenocytes were prepared. **A.** Flow cytometric analysis of Th17, Th1, Th2 and Treg cells from one representative experiment. Single cell suspensions of splenocytes were stimulated with PMA/ionomycin in the presence of Golgistop for 6 h, followed by surface staining with anti-CD3-APC and anti-CD4-FITC and intracellularly staining with PE-conjugated antibodies against IL-17A, IFN-γ, IL-4 or isotype IgG2a control antibody for FACS analysis of Th17, Th1 or Th2 cells. Splenocytes were also stained with the Mouse Regulatory T Cell Staining Kit for detection of Treg cells. Cells were gated on the CD3^+^ population for analysis of Th17, Th1, Th2 cells analysis, or gated on the CD3^+^CD4^+^ population for analysis of Treg cells. **B.** The proportions of Th17, Th1, Th2 and Treg cells in CD4^+^ T cells. Results are expressed as mean ± SD of eight mice from two independent experiments. *P<0.05; **P<0.01; ***P<0.001, compared to control mice injected with PBS or isotype antibody. **C.** ELISA detection of cytokines in the supernatant produced by splenocytes from *S. japonicum* infected mice after administration of rmIL-17A or anti-IL-17A neutralizing mAb. Splenocytes (2×10^5^/well in 200 µl of complete media) were cultured in 96 well plates with PMA/ionomycin. Culture supernatants were collected after 72 h, and cytokines were detected by ELISA. Results are expressed as mean ± SD of eight mice from two independent experiments. *P<0.05; **P<0.01; ***P<0.001, compared to control mice injected with PBS or isotype antibody. SEA (**D**) and SWA (**E**) specific IgG, IgG1 and IgG2a antibodies in serum from *S. japonicum* infected mice after administration of rmIL-17A or anti-IL-17A neutralizing mAb were detected by ELISA. Results are expressed as mean ± SD of eight mice from two independent experiments. *P<0.05; **P<0.01; ***P<0.001, compared to control mice injected with PBS or isotype antibody.

To further investigate the possible mechanism underlying the effects of IL-17 on the response to anti-schistosome infection and the hepatic immunopathology, we detected the levels of CD4^+^T cells, cytokines and antibody responses in mice after administration of rmIL-17A or anti-IL-17A neutralizing mAb. The proportions of Th1/Th2/Th17/Treg cells did not change after injection with either rmIL-17A to increase the level of IL-17 or anti-IL-17A neutralizing mAb to decrease the level of IL-17 *in vivo* (Fig. 7A and 7B). The production of IFN-γ, IL-4, IL-6, IL-23, IL-21, TGF-β and IL-17 from splenocytes of *S. japonicum* infected mice increased after injection with rmIL-17A (Fig. 7C). However, injection of *S. japonicum* infected mice with anti-IL-17A neutralizing mAb resulted in the increase of IFN-γ, IL-4, IL-6 and IL-21, but the decrease of TGF-β and IL-17 produced by mouse splenocytes. When compared to the PBS control group, administration of rmIL-17A statistically significantly decreased the level of SEA specific IgG1 antibody but increased the level of SWA specific IgG2a antibody in *S. japonicum* infected mice (Fig. 7D and 7E). However, when compared to the isotype antibody control group, administration of anti-IL-17A neutralizing mAb statistically significantly increased the levels of SEA specific IgG1, IgG2a and total IgG antibodies, as well as SWA specific IgG2a antibody in *S. japonicum* infected mice.

## Discussion

During the past several years, the Th1/Th2 paradigm has been updated to include a third helper subset called Th17. Through the induction of chemokines and the recruitment of other effector T cell populations [53]–[55], the responses of Th17 cells dominate in response to certain defined pathogens and play important roles in both host defense against pathogens and immunopathogenesis [11], [56]. Schistosomiasis is a typical chronic infectious disease. Infection induces the generation of Th1, Th2 and Treg cells, as well as Th17 cells that are involved in the formation of hepatointestinal perioval granulomas. In this study, we investigated the kinetics of the generation of Th17 cells induced by parasite antigens from different stages of *S. japonicum* infection in mice as well as the role of Th17 cells in the host protective responses.

In our study, as the parasites began to produce eggs, the granulomas formed in the mouse liver and developed continuously. After the eggs were deposited into the liver and the granulomas were beginning to form, the proportion of Th17 cells in the spleen, mesenteric lymph nodes and liver CD4^+^ T cells increased slowly up to five weeks post-infection but then increased more rapidly between five and eight weeks post-infection while accompanied by the development of the granulomas. Meanwhile, the proportions of Th1, Th2 and Treg cells in the CD4^+^ T cells also increased. These findings suggested that the schistosomal antigens induced the simultaneous generation of both Th17 cells and the other CD4^+^ subsets that are thought to suppress the generation of Th17 cells during infection as reported in many previous studies [2], [5], [32]; however, these factors seem to have failed to suppress the generation of Th17 cells in our *S. japonicum* infection experiments. In addition, the results also suggested it may be the egg antigens of *S. japonicum* that were responsible for the more rapid increase in the proportion of Th17 cells in total CD4^+^ T cells from five weeks onward after infection.

Schistosome eggs and adult worms are two important sources of antigens exposed to the host during *S. japonicum* infection [57]. They both have the potential to induce Th1, Th2, Th17 and Treg cells and the corresponding cytokines. Therefore, we confirmed the above hypothesis by using to SEA or SWA to immunize mice as well as to stimulate CD4^+^ cells *in vitro*, and our data showed that it was SEA that preferentially induced the generation of Th17 cells and production of IL-17.

The generation and suppression of Th17 cells by Th1, Th2 and Treg cells and/or their cytokines have been demonstrated in numerous studies of *in vivo* and/or *in vitro* induction of T cells under defined polarizing conditions [25], [27], [32]–[34]. Based on these reports, the current widely accepted differentiation mode of the Th17 cell subset is as follows. In the presence of TGF-β and IL-6, CD4^+^ T cells are induced to express the transcription factor RORγt by stimulating the STAT3 and Smad signaling pathway, which leads to the differentiation of Th17 cells. IL-21 together with TGF-β also stimulate the alternative pathway for Th17 differentiation. Meanwhile, the mature Th17 cells amplify themselves by autocrine IL-21. IL-23 also contributes to the maintenance of Th17 cell stability through engagement of the IL-23R, which is expressed by memory or activated T cells [58]. Simultaneously, IFN-γ and IL-4 can effectively inhibit the generation of Th17 cells [2], [5], [59].

Our study clearly showed that during the course of *S. japonicum* infection, in parallel with the increase of the proportion of Th17 cells, both the inducing (TGF-β, IL-6, IL-21 and IL-23) and inhibitory (IFN-γ, IL-4, Th1, Th2 and Treg cells) factors of Th17 cell generation increased as the infection progressed. These results suggested that a multicellular pathogen such as *S. japonicum* introduced complex sets of antigens at different stages of infection into the host that could promote both the inducible and the inhibitory factors of Th17 cell generation, but the overall net result was an increase in Th17 cells. In another words, the observed increase in Th17 cells during *S. japonicum* infection was probably due to the ability of the *S. japonicum* antigens to more strongly upregulate Th17 inducing factors than the Th17 suppressive factors. In addition, immunization of mice with SEA also preferentially induced Th17 cell generation and the production of higher levels of known factors involved in the generation of Th17 cells (TGF-β, IL-23 and IL-21), while accompanied by the increase in the reported inhibitory factors of Th17 cell generation (Treg and Th2 cells, as well as IL-4).

Many studies have shown that Th17 cells play important roles both in host defenses against extracellular pathogens [6], which are not efficiently cleared by Th1-type and Th2-type immunity and in immunopathogenesis caused by infection. In *S. japonicum* infection, it has been reported that Th17 may play an important role in the liver immunopathogenesis and in the formation and growth of granulomas around the eggs produced by the adult worm. The findings indicate that the development of severe murine schistosomiasis correlates with high levels of IL-17 and suggest that the exacerbated egg-induced immunopathology is largely mediated by the subset of SEA-induced Th17 cells which produces IL-17 [12], [13]. Our study also suggested that the IL-17 level was positively related to the severity of liver pathogenesis, which was possibly due to the enrichment of inflammatory cells including neutrophils and eosinophils in the granulomas. However, there has been no evidence reported yet to indicate whether Th17 and its product IL-17 either improve or impair the anti-schistosome immune response. Considering that the protection against *S. japonicum* infection is mainly based on the clearance of the schistosomulum at the early stage of infection, we investigated the potential protective effect of IL-17 levels during that period. Our results showed that administration of rmIL-17A to mice failed to reduce the worm and egg burdens, indicating that high levels of IL-17 did not contribute to the protective responses. Instead, decreasing the level of IL-17 in mice by injecting anti-IL-17A neutralizing mAb led to reductions of worm and egg burdens, suggesting that the decrease of IL-17 levels contributed to effective protective responses against *S. japonicum* infection. Our study further showed that administration of the anti-IL-17A neutralizing mAb increased the levels schistosome specific IgG1, IgG2a and/or IgG, suggesting that increased antibody-dependent-cell-cytoxicity (ADCC), one of the well accepted mechanisms of killing extra-cellular residing pathogens including schistosome [60], may at least partially contributed to the more effective protective responses against *S. japonicum* infection. However, the mechanism underlying the role of IL-17 in the protection against *S. japonicum* infection needs to be further investigated.

In summary, for the first time our study reported on the kinetics of the generation of Th17 cells, which were likely preferentially induced by egg antigens, during *S. japonicum* infection. We also determined that the proportion of Th17 cells increased together with other CD4^+^ subsets reported to inhibit them, including Th1, Th2 and Treg cells, as well as their suppressive cytokines in a *S. japonicum* infection mouse model. These findings suggest that the generation of Th17 cell is determined by the integrated impact of the inducing and suppressive factors promoted by parasitic antigens. More importantly, our study for the first time indicates that a decrease in the level of IL-17 in the early stage of *S. japonicum* infection may contribute to the host protective responses.

## Supporting Information

Figure S1 **SEA-specific Th17 responses in** ***S. japonicum*** **infected mice.** For each of two independent experiments, 6 female C57BL/6 mice were infected with 12 cercariae of *S. japonicum* per mouse. After eight weeks, single cell suspensions of splenocytes, lymphocytes or liver cells were *in vitro* stimulated with SEA or PBS for 48 h. **A.** Cells were surface stained with anti-CD3-APC and anti-CD4-FITC and then intracellularly stained with PE-conjugated antibodies against IL-17A or isotype IgG2a control antibody for FACS analysis of SEA-specific Th17 cells. Data were from one representative experiment and cells were gated on the CD3^+^ population. **B.** The percentages of SEA-specific Th17 cells in total CD4^+^ T cells from mouse spleens, mesenteric lymph nodes and livers. Data are expressed as the mean ± SD of 12 mice from two independent experiments. **P<0.01, compared to PBS control group. **C.** The culture supernatants were collected after 48 h of SEA *in vitro* stimulation for detection of IL-17 by ELISA. Data are expressed as the mean ± SD of 12 mice from two independent experiments. *P<0.05; **P<0.01, compared to PBS control group.
(TIF)

Figure S2 ***In vitro*** **induction of CD4^+^T cell differentiation by SEA or SWA.** Using a negative selection cell isolation kit, CD4^+^ T cells were isolated from naïve mouse splenocytes in the negative fraction, while APCs were obtained from the positive fraction and irradiated with 30 Gy. The CD4^+^ T cells (2×10^6^/well) were cultured in triplicate wells of 24-well plates with APCs (1×10^6^/well) in the presence of 50 µg/ml of SEA, SWA or PBS as control in complete RPMI 1640 medium (2 ml/well). After 72 h, the cells were surface stained with anti-CD3-APC and anti-CD4-FITC, and then intracellularly stained with PE-conjugated antibodies against IL-17A, IFN-γ, IL-4 or isotype IgG2a control antibody for FACS analysis of Th17, Th1 or Th2 cells. Splenocytes were also stained with the Mouse Regulatory T Cell Staining Kit for Treg cell detection. **A.** Proportions of Th17, Th1, Th2 and Treg cells in CD4^+^ T cells. Results are expressed as mean ± SD of nine samples from three independent experiments. *P<0.05; **P<0.01; ***P<0.001, compared to PBS control. **B.** Flow cytometric analysis from one representative experiment. Cells were gated on the CD3^+^ population for analysis of Th17, Th1 and Th2 cells or gated on CD3^+^CD4^+^ population for analysis of Treg cells.
(TIF)
