# Supplementary figures and images for: Dynamics of Th17 Cells and Their Role in Schistosoma japonicum Infection in C57BL/6 Mice
Source: PLoS Negl Trop Dis. 2011 Nov 15;5(11):e1399. doi: 10.1371/journal.pntd.0001399 (PMC3216943; doi:10.1371/journal.pntd.0001399)

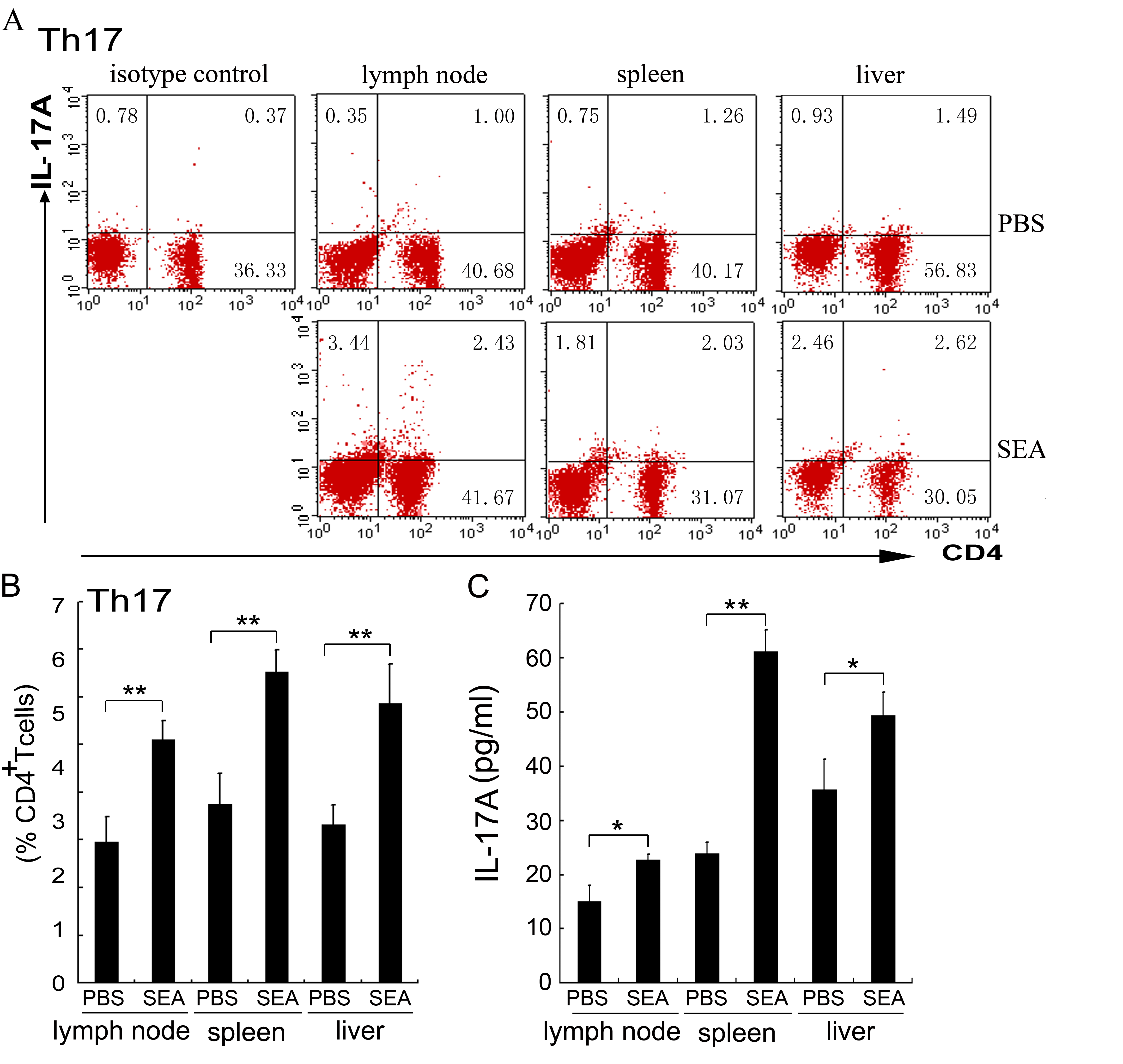

Supplement: Figure S1 — SEA-specific Th17 responses in S. japonicum infected mice. For each of two independent experiments, 6 female C57BL/6 mice were infected with 12 cercariae of S. japonicum per mouse. After eight weeks, single cell suspensions of splenocytes, lymphocytes or liver cells were in vitro stimulated with SEA or PBS for 48 h. A. Cells were surface stained with anti-CD3-APC and anti-CD4-FITC and then intracellularly stained with PE-conjugated antibodies against IL-17A or isotype IgG2a control antibody for FACS analysis of SEA-specific Th17 cells. Data were from one representative experiment and cells were gated on the CD3+ population. B. The percentages of SEA-specific Th17 cells in total CD4+ T cells from mouse spleens, mesenteric lymph nodes and livers. Data are expressed as the mean ± SD of 12 mice from two independent experiments. **P<0.01, compared to PBS control group. C. The culture supernatants were collected after 48 h of SEA in vitro stimulation for detection of IL-17 by ELISA. Data are expressed as the mean ± SD of 12 mice from two independent experiments. *P<0.05; **P<0.01, compared to PBS control group. (TIF) [file pntd.0001399.s001.tif]

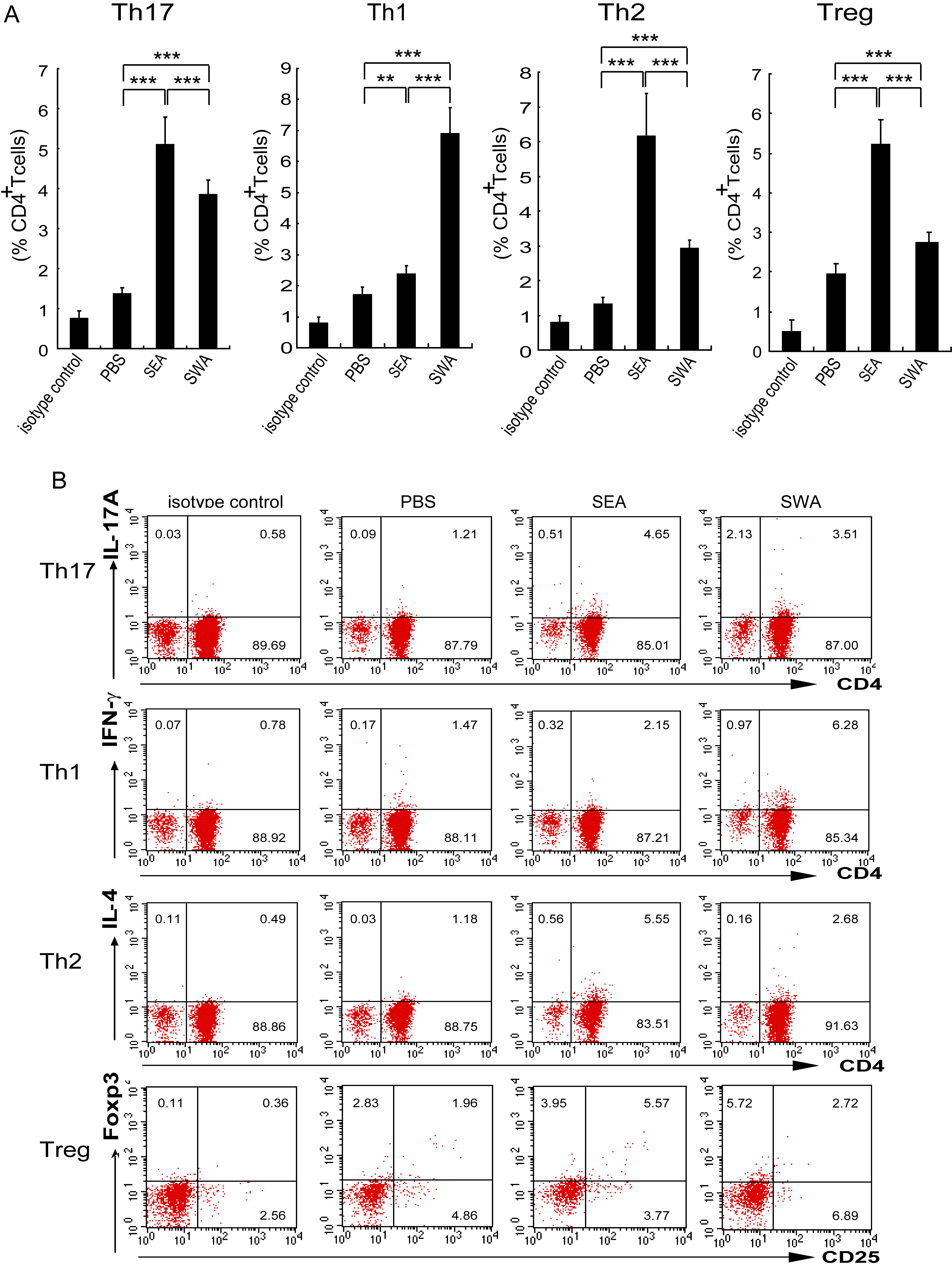

Supplement: Figure S2 — In vitro induction of CD4+T cell differentiation by SEA or SWA. Using a negative selection cell isolation kit, CD4+ T cells were isolated from naïve mouse splenocytes in the negative fraction, while APCs were obtained from the positive fraction and irradiated with 30 Gy. The CD4+ T cells (2×10^6^/well) were cultured in triplicate wells of 24-well plates with APCs (1×10^6^/well) in the presence of 50 µg/ml of SEA, SWA or PBS as control in complete RPMI 1640 medium (2 ml/well). After 72 h, the cells were surface stained with anti-CD3-APC and anti-CD4-FITC, and then intracellularly stained with PE-conjugated antibodies against IL-17A, IFN-γ, IL-4 or isotype IgG2a control antibody for FACS analysis of Th17, Th1 or Th2 cells. Splenocytes were also stained with the Mouse Regulatory T Cell Staining Kit for Treg cell detection. A. Proportions of Th17, Th1, Th2 and Treg cells in CD4+ T cells. Results are expressed as mean ± SD of nine samples from three independent experiments. *P<0.05; **P<0.01; ***P<0.001, compared to PBS control. B. Flow cytometric analysis from one representative experiment. Cells were gated on the CD3+ population for analysis of Th17, Th1 and Th2 cells or gated on CD3+CD4+ population for analysis of Treg cells. (TIF) [file pntd.0001399.s002.tif]
